# Health Implications of Occupational Exposure of Butchers to Emissions from Burning Tyres

**DOI:** 10.29024/aogh.2321

**Published:** 2018-08-31

**Authors:** Francis O. Okonkwo, Anoka A. Njan, Chukwunonso E.C.C. Ejike, Uchechukwu U. Nwodo, Ikechukwu N.E. Onwurah

**Affiliations:** 1 Environmental and Occupational Toxicology Unit, Department of Biochemistry, Plateau State University, Bokkos, Plateau State, NG; 2 Department of Biochemistry, University of Nigeria, Nsukka, Enugu State, NG; 3 Department of Pharmacology and Therapeutics, Faculty of Basic Health Sciences, University of Ilorin, Ilorin, Kwara State, NG; 4 Department of Medical Biochemistry, Alex Ekwueme Federal University, Ndufu-Alike, Ebonyi State, NG; 5 Department of Biochemistry and Microbiology, Forth Hare University, Private Bag X1314, Alice 5700, ZA

## Abstract

**Background:**

Flames from burning scrap tyres are used in de-furring animals for human consumption in most parts of Nigeria. Emissions from tyres are known to contain a myriad of toxic mixtures especially particulate matter (PM), volatile organic compounds, hazardous air pollutants, and inspirable metals, some of which are known human carcinogens. This cross-sectional study investigated the deleterious health effects of these emissions in occupationally-exposed workers at the Dei-Dei Abattoir, Abuja, Nigeria.

**Methods:**

A total of 156 respondents were divided into two groups. Group 1 (124 butchers) and group 2 [32 administrative staff (AS)]. Data from digital spirometry were used to determine the association between chronic exposure to tyre emissions and lung function. Urinary 1-Hydroxypyrene concentration, phenolic compounds levels and heavy metal concentrations were determined. Also ambient PM and polycyclic aromatic hydrocarbons (PAHs) concentrations at 3 delineated points in the abattoir were measured.

**Findings:**

Spirometry results showed significant deterioration of lung function in the butchers. The concentration of 1-Hydroxypyrene (μg/molCret) in the post-shift urine samples of the butchers was significantly higher (P < 0.05) in butchers relative to the AS (0.52 ± 0.13 Vs 0.20 ± 0.07, respectively). Similarly the concentrations of zinc and nickel (mg/l) were significantly higher in the butchers compared to the AS (zinc: 0.91 ± 0.19 Vs 0.31 ± 0.28, respectively; nickel: 0.11 ± 0.06 Vs 0.06 ± 0.02, respectively). Anthracene, fluoranthene, pyrene, benzo-a- pyrene, and PM concentrations were significantly higher at the de-furring point when compared to the wash bay and the administrative building, especially between 8.00 and 8.30 am.

**Conclusion:**

Occupational exposure to scrap tyre emissions resulted in significant adverse health effects. The existing laws banning the use of burning tyres in meat processing should be enforced while the use of personal protective equipment should be encouraged in abattoirs.

## Introduction

Management of pollution caused by end-of-life tyres (ELTs) is a major problem, especially in developing countries like Nigeria. In developed economies, over 300 million scrap tyres are generated annually [1,2]. They are largely converted to healthy and environmentally friendly uses in energy generation, civil engineering, devulcanization and production of organic materials by pyrolysis [2]. In Nigeria and other developing countries, lack of technological know-how, costs and many other factors may be attributed to the different scenario experienced. In these countries, when tyres are not burnt in waste disposal bins, they are used in bonfires to protest real or perceived injustices from the government/authorities during civil unrests, and in some places, they are used during festivities ushering in the new year.

Tyres are made from natural and synthetic rubber polymers; oil fillers; sulfur and sulfur compounds; phenolic resins; clay; aromatic, naphthenic, and paraffinic oils; carbon black; zinc oxide; titanium dioxide; fatty acids; and fibers made from steel, nylon, polyester or rayon [3,4]. When tyres burn, especially in the open, several compounds and particles are released into the environment. They include criteria pollutants, including particulates such as carbon monoxide (CO), oxides of sulphur (SO_x_), oxides of nitrogen (NOx) and volatile organic compounds (VOCs), and noncriteria hazardous air pollutants (HAPs), such as polychlorinated biphenyls (PCBs), dioxins, furans, hydrogen chloride, benzene and polycyclic aromatic hydrocarbons (PAHs) [1]. Metals, such as arsenic, cadmium, nickel, zinc, mercury, chromium and vanadium, are also released into the environment during the process [5,6]. All these emissions are usually adsorbed to, and released into, the environment as particulate matter (PM).

Air emissions from burning tyres have been shown to be more toxic than those of a combustor, regardless of fuel source [1,7]. At the turn of the millenium, when the World Health Organization (WHO) issued its second air quality guidelines for Europe [8], there was an upsurge in research into the health effects of airborne PM. The research results have deepened our understanding of the risks posed to the environment and, ultimately. to human health by uncontrolled exposure to airborne PM. Adverse health outcomes subsequent to airborne PM exposure have been established even at low concentrations such that further residual risk assessment and characterization using background concentration levels are often warranted [9].

The implication is that exposure to these emissions constitutes significant acute and chronic risks and hazards to individuals exposed to such pollutants, either occupationally or as a consequence of where they live. The acute and chronic risks due to exposure to emissions from tyre fires have been implicated in health effects, including irritation of the eyes, skin, and mucous membranes; depression of the central nervous system; exacerbation of respiratory and cardiac conditions and some cancers [1]. Apart from developed countries that monitor ELTs, countries in sub-Saharan Africa, South America and Southeast Asia have poor data procurement and management systems such that it is difficult to ascertain exactly what is being emitted, the quantity being emitted and the extent of the danger to human health, especially for children and the elderly [1].

In Nigeria and some West African countries, burning tyres are used to defur slaughtered animals. In abattoirs all over Nigeria, tyres, plastics and other incendiary materials are burnt without considering the health and toxicological impact on workers and the general public. The effect on the immediate environment is also scarcely considered. Therefore, the aim of this study was to investigate possible adverse health effects on workers (butchers/rosters and administrative staff) at the Dei-Dei Abattoir and Livestock Market in Abuja who are exposed to various degrees of emissions from burning tyres used in the de-furring of animals.

## Materials And Methods

### Study Location

**Figure 1:**
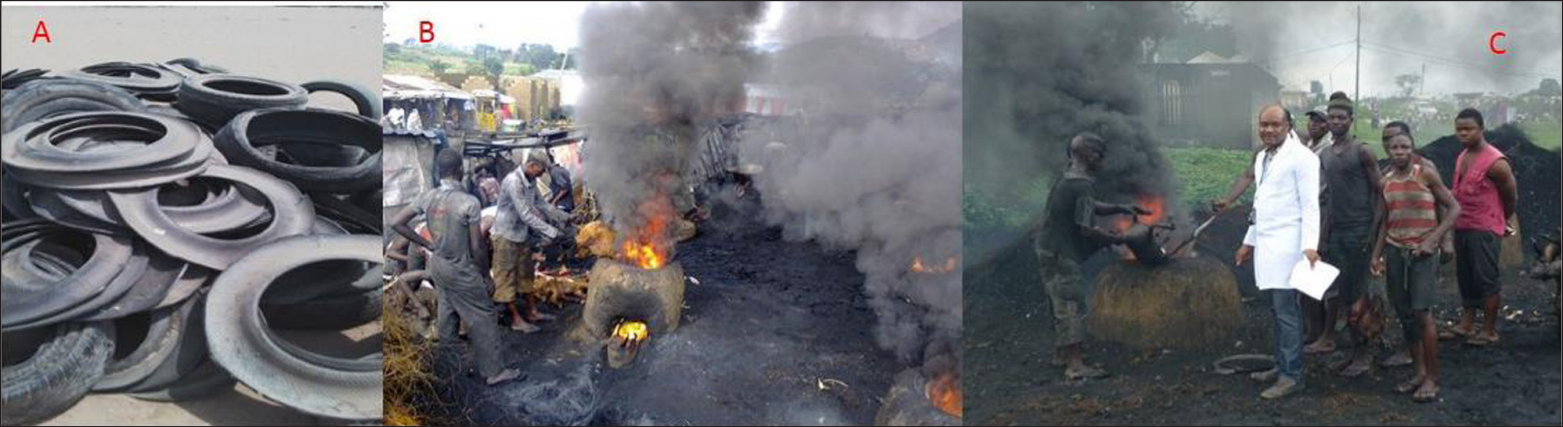
Scrap tyres and de-furring mounds utilized by butchers in meat processing at Dei-Dei abattoir, Abuja. **(A)** Scrap tyres ready for use in the abattoir. **(B)** De-furring mound for processing slaughtered animals with scrap tyres. **(C)** Butchers de-furring goat with F.O.O. inset.

Dei-Dei Abattoir and Livestock Market in Abuja Municipal Area Council of the Federal Capital Territory, Nigeria, was the site of this study. Housing two big slaughterhouses and employing over three hundred people as butchers, livestock sellers and management staff—including veterinary and administrative staff—from the local government area, the abattoir is the biggest in the Federal Capital Territory (FCT). The butchers use mud blocks to erect fire mounds with a round base and tapering top covered with metal gauze where the animal parts to be defurred are placed (Figure 1). Burning tyres used for this purpose are placed inside the fire mound through a hole on the side, which also serves as an air inlet.

### Study Population

Ethical approval (number FHREC/2013/01/10/16-05-13) was sought and obtained from the Federal Capital Territory Human Health Research Ethics Board. The rationale and methods for the study were explained to the abattoir workers. Thereafter, participant information sheet and informed consent forms (PISICF) were administered to the workers in the abattoir prior to the commencement of the study. Standard questionnaires were also administered to the workers and the general public to ascertain their level of awareness of the subject matter. A total of 124 butchers and 32 administrative staff gave written informed consent and were recruited. Purposive sampling [10] and mixed sampling techniques were incorporated to increase depth of understanding of the subject matter while improving reliability and validity of the findings [11,12]. Only workers who had worked at the abattoir for three or more years were included in the study. Participants were free to withdraw from the study at any point.

### Experimental Design

The 156 subjects recruited were divided into two groups. Group 1 consisted of 124 butchers, who served as test subjects, while Group 2 was made up of 32 administrative staff (AS), who served as control subjects. The experimental design for this study was based on the work of Wang and colleagues [13], involving the indirect measurement of environmental parameters and the direct measurement of human biomarkers of exposure and/or effect. The environmental parameters measured were PM and some priority PAHs in fumes from burning tyres. The human biomarkers measured include urinary concentration of 1-hydroxypyrene, some heavy metals and phenolic compounds.

### Delineation of Points for Airborne PM and PAHs Measurements

Three sampling points were used: A, B and C. Point A, at latitude 9.12027778 and Longitude 7.26472222, corresponded to actual defurring point. Point B, at latitude 9.11777778 and longitude 7.25722222, corresponded to 50 metres away from Point A and served as a wash-bay. Point C, at latitude 9.07666667 and longitude 7.44500000, corresponded to 200 metres away from Point A and served as offices for administrative staff.

### Measurement of Environmental Parameters

Aerocet 531S (Met One Instruments, Texas, USA) was used to assess the PM concentration from the 3 delineated points at randomly chosen moments between 7 a.m. and 9 a.m. for 14 days. Each sampling run was for 180 seconds (3 minutes), and the results were automatically stored for future retrieval. The parameters measured by this equipment include PM_1_, PM_2.5_, PM_7_, PM_10_ (defined as particles with aerodynamic diameter ≤ 1, 2.5, 7, and 10 mm respectively) and total suspended particles (TSP).

Eight priority PAHs (Sigma-Aldrich, Taufkirchen, Germany), namely anthracene, fluoranthane, pyrene, indeno-(123) c,d-pyrene, benzo-e-pyrene, benzo-b-fluranthane, benzo-a-pyrene and dibenz-ah-anthracene in ambient air in and around the abattoir, especially at the 3 delineated points, were sampled at the breathing zone of the roasters using Universal PCXR4 automated personal air sampler (SKC Inc, Pittsburgh, Pennsylvania, USA) at a flow rate of 2 L/minute (120 L/hour), following the manufacturer’s instructions [14].

### Lung Function Measured by Spirometry

The lung function of the subjects was measured using standard methods [15,16] to determine the peak expiratory flow (PEF), which is the maximum flow generated during expiration performed with maximal force and started after a full inspiration [17]; forced expiratory volume (FEV), which is usually the volume exhaled during the first second of a forced expiratory manoeuvre; forced vital capacity (FVC), which is the volume change of the lung between a full inspiration to total lung capacity and a maximal expiration to residual volume [17]; and the ratio of the FEV/FVC (expressed as a percent). All these values were automatically generated. The highest values, following a minimum of eight attempts, were recorded per subject [15]. Spirometry was conducted on the respondents at the beginning of each quarter.

### Urine Samples and Other Anthropometric Measurements

On sampling days, post-shift urine samples were collected in amber plastic containers from the butchers and administrative staff at the end of the day’s work. The urine samples were stored on ice and transported to the laboratory. The samples were promptly transferred to a freezer and stored at –20°C pending further analysis. The analyses carried out on the post-shift urine samples include the concentration of urinary 1–Hydroxypyrene (1-OHP), determined using the methods of Jongeneelen *et al.* [18] and adapted to local laboratory conditions; urinary heavy metal load, determined using the methods of the American Public Health Association (APHA) [19]; and total phenolic compounds, determined using the method of Yamaguchi and Hayashi [20].

The systolic and diastolic blood pressures and pulse rates of the subjects were measured between 7 a.m. and 8 a.m. on sampling days using an oscillometric device. Subjects were required to be seated for 10 minutes before measurements were taken. Three separate measurements were taken per subject, each after 5 minute intervals, and the average of the last two were recorded. Other parameters, such as age, height and weight were measured following standard protocols. Body mass index (BMI) was calculated as weight (kg)/height (m^2^).

### Statistical Analysis

Quantitative data were reported as mean ± standard deviation, and differences between the mean values for butchers and the administrative staff were compared with the student’s t-test. Categorical variables were reported as percentages, and differences between them were tested for significance using the Chi squared test and the Fisher’s exact test. In all statistical tests, a significance threshold of *p* < 0.05 was adopted. Data analysis was carried out using IBM-SPSS version 20.0 (IBM Corp. Atlanta, GA).

## Results

### Data from Distributed Questionnaires

Detailed results of the questionnaire administered on defurrers and some other respondents living and/or working in and around the abattoir are summarized in Table 1.

**Table 1:** Baseline sociodemographic characteristics of workers and adjoining residents of Dei-Dei Abattoir and Livestock Market, Abuja, Nigeria (N = 200).

Parameter/Variable	n (%)

Sex of Respondents	
Male	137 (68.5)
Female	63 (31.5)
Age Distribution of Respondents (Years)	
11–20	5 (2.5)
21–30	112 (56.0)
31–40	53 (26.5)
41–50	20 (10)
>50	10 (5.0)
Occupation of Respondents	
Butchers	136 (68.0)
Students	10 (5.0)
Civil/Public Servants	38 (19.0)
Livestock Dealers/Businessmen	16 (8.0)
Average Income of Respondents/day (Nigerian Naira)*	
0–500	36 (18.0)
501–1000	74 (37.0)
1001–2000	51 (25.5)
2001–3000	24 (12.0)
>3000	15 (7.5)
Length of Time in Current Employment	
<1	21 (10.5)
1	30 (15.0)
2	37 (18.5)
3	38 (19.0)
4	29 (14.5)
5	14 (7.0)
≥6	31 (15.5)
Respondents with Safety Training	
Yes	93 (46.5)
No	107 (53.5)
Respondents’ Use of PPEs**	
Yes	27 (13.5)
No	173 (86.5)
Respondents Smoking for as long as 1 year	
Yes	25 (12.5)
No	175 (87.5)
Alcohol use by Respondents	
Yes	33 (16.5)
No	167 (83.5)
Common Symptoms Experienced by Respondents in the Preceding 2 weeks	
CP + DB + CH + CI + BS	89 (44.5)
CP + DB + BS	65 (32.5)
BS + CI	32 (16.0)
BS	14 (7.0)

*Naira is Nigerian local currency and exchanges to the USD @ approx. N370 to $1; **PPEs – Personal Protective Equipment; CP – Chest pain; DB – Difficulty breathing; CH – Cough; CI – Compromised Immunity; BS – Breathlessness.

### Outcome of Anthropogenic Measurements

Table 2 shows the butchers were significantly younger than the administrative staff (30.1 ± 6.2 versus 42.5 ± 10.1 years; *p* < 0.05). They were also taller, weighed less and had significantly lower mean BMI values (23.4 ± 4.6 versus 29.9 ± 3.5 kg/m^2^; *p* < 0.05) relative to the control.

**Table 2:** Anthropometric measurements in butchers and administrative staff of Dei-Dei Abattoir, Abuja, Nigeria.

*Parameter*	Butchers (N = 156)	Admin Staff (N = 32)	*P* (2-tailed)	

Age (years)	31.0 ± 6.2	42.5 ± 10.13	0.017	*
Height (cm)	171.4 ± 4.6	161.8 ± 4.5	0.001	*
Weight (kg)	70.1 ± 16.5	77.5 ± 12.7	0.369	
BMI (kg/m2)	23.7 ± 1.32	29.8 ± 3.5	0.016	*
Pulse rate (beats/min)	75.1 ± 12.1	87.3 ± 13.5	0.090	
SBP (mm/Hg)	122.3 ± 17.3	145.0 ± 28.8	0.090	
DBP (mm/Hg)	78.2 ± 13.5	91.3 ± 19.7	0.147	

*Statistically significant at *p* < 0.05. SBP and DBP represent systolic blood pressure and diastolic blood pressure, respectively.

### Concentrations of Airborne PM with Respect to Time and Delineated Points

Table 3 shows the result of particulate matter (PM_1_, PM_2.5_, PM_7_, PM_10_ and TSP) measured at points A, B and C at varying times during the work day in the abattoir. The concentrations of PM_1_, PM_2.5_, PM_7_, PM_10_ and TSP generally were low at 7 a.m. but increased with time until 8.30 a.m., after which they dropped by 9 a.m., irrespective of the point of sampling. The differences in the concentrations of PM_1_ at all three sampling points at 7 a.m. were statistically similar (*p* > 0.05) compared to the other time points (except 8 a.m. at point B). For PM_2.5_, PM_7_, PM_10_ and TSP, significant differences in their concentrations at the different time points compared to 7 a.m. were found only at point A. At point A, the differences in the concentrations of PM_2.5_, PM_7_, PM_10_ and TSP at 7 a.m. compared to 8 a.m. and 8.30 a.m. were all significant (*p* < 0.05), except for PM_2.5_ at 8 a.m. At 9 a.m, the concentrations were statistically similar (*p* > 0.05) to the concentrations at 7 a.m. Clearly significant variations in the concentrations of the particulate matter with time were found only at the defurring point (point A). Their concentrations at the wash area (point B) and the administrative building (point C) were statistically similar at all the time points studied.

**Table 3:** Particulate matter measured in Dei-Dei Abattoir between 7:00 and 9:00 a.m.

Location	Time (am)	PM_1_ (×10^3^ μg/cm^3^)	PM_2.5_ (×10^3^ μg/cm^3^)	PM_7_ (×10^3^ μg/cm^3^)	PM_10_ (×10^3^ μg/cm^3^)	TSP (×10^3^ μg/cm^3^)

Point A	7:00	0.0267 ± 0.02	0.0637 ± 0.04	0.3537 ± 0.42	0.4327 ± 0.55	1.2620 ± 0.71
	8:00	0.1597 ± 0.09	0.3947 ± 0.18	**4.1247 ± 3.34	**4.8980 ± 4.05	**5.7260 ± 4.62
	8:30	0.0697 ± 0.03	**1.7463 ± 2.59	**4.7200 ± 4.34	**5.5387 ± 4.75	**6.2900 ± 5.00
	9:00	0.0913 ± 0.09	0.2490 ± 0.25	0.3353 ± 0.29	0.3607 ± 0.26	0.6440 ± 0.58
Point B	7:00	0.0373 ± 0.02	0.1750 ± 0.18	0.3790 ± 0.20	0.4720 ± 0.29	0.9690 ± 0.99
	8:00	*0.2370 ± 0.32	0.1487 ± 0.07	0.2647 ± 0.13	0.3150 ± 0.13	0.3720 ± 0.14
	8:30	0.0393 ± 0.03	0.0853 ± 0.07	0.1240 ± 0.09	0.1423 ± 0.09	0.1717 ± 0.08
	9:00	0.0390 ± 0.03	0.0617 ± 0.04	0.1990 ± 0.23	0.3127 ± 0.41	0.5850 ± 0.84
Point C	7:00	0.2217 ± 0.19	0.3817 ± 0.52	0.7067 ± 0.76	0.7767 ± 0.80	1.1923 ± 1.33
	8:00	0.0503 ± 0.01	0.1227 ± 0.05	0.2083 ± 0.07	0.2667 ± 0.09	0.3843 ± 0.25
	8:30	0.0463 ± 0.03	0.4047 ± 0.51	0.1740 ± 0.14	0.2117 ± 0.12	0.3487 ± 0.18
	9:00	0.0353 ± 0.03	0.0783 ± 0.06	0.2507 ± 0.19	0.3160 ± 0.27	0.4340 ± 0.43

** = *p* < 0.01; * = *p* < 0.05; comparisons are made to values for 7 a.m.

### Polycyclic Aromatic Hydrocarbons in Air Samples from the Delineated Points

The concentrations of all the PAHs in the air samples were highest at point A and lowest at point C (Table 4). Other than indeno(1,2,3-cd)pyrene (and benzo(b)fluoranthene at point B relative to point A), the other PAHs had concentrations in samples from point A that were significantly higher (*p* < 0.05) than concentrations found in points B and C (Table 4).

**Table 4:** Concentrations of some polycyclic aromatic hydrocarbons (PAHs) at Dei-Dei Abattoir, Abuja.

Location	NAPHTH (μg/cm^3^)	ANTHR (μg/cm^3^)	FLUOR (μg/cm^3^)	PYR (μg/cm^3^)	IND-123 (μg/cm^3^)	BENZ-b-FL (μg/cm^3^)	BENZ-A-P (μg/cm^3^)

Point A	0.79 ± 0.02	0.05 ± 0.00	0.32 ± 0.01	0.03 ± 0.00	0.03 ± 0.02	0.05 ± 0.00	0.07 ± 0.01
Point B	**0.56 ± 0.04	**0.03 ± 0.00	**0.21 ± 0.01	*0.03 ± 0.01	0.04 ± 0.00	0.05 ± 0.00	**0.06 ± 0.01
Point C	**0.21 ± 0.01	**0.02 ± 0.00	**0.16 ± 0.00	**0.02 ± 0.00	0.03 ± 0.00	**0.04 ± 0.00	**0.02 ± 0.02

** = *p* < 0.01; * = *p* < 0.05; comparisons are made to Point A. NAPHTH – Naphthalene; ANTHR – Anthracene; FLUOR – Fluoranthene; PYR – Pyrene; IND-123 – Indeno(1,2,3-cd)pyrene; BENZ-b-FL – Benzo-b-fluoranthene; BENZ-A-P – Benzo-a-pyrene.

### Lung Function Spirometric Parameters

As seen in Table 5, the mean PEV was significantly lower (*p* < 0.05), while the mean FEV and FVC were significantly higher (*p* < 0.05), in the butchers compared to the administrative staff.

**Table 5:** Lung function parameters of butchers and administrative staff at Dei-Dei Abattoir, Abuja, measured over four quarters.

Parameter	Q1		Q2		Q3		Q4	

PEV-Butchers	250.17 ± 26.89	*	233.50 ± 40.10	*	241.83 ± 31.84	*	256.00 ± 23.59	*
PEV- AS	571.67 ± 54.43		571.67 ± 54.43		480.00 ± 94.78		570.00 ± 54.75	

FEV- Butchers	2.42 ± 0.36	*	2.53 ± 0.50	*	2.32 ± 0.41	*	2.38 ± 0.39
FEV-AS	2.05 ± 0.22		2.06 ± 0.21		2.14 ± 0.30		2.08 ± 0.19	

FVC-Butchers	3.13 ± 0.50	*	3.30 ± 0.82	*	3.13 ± 0.50	*	3.17 ± 0.51	*
FVC-AS	2.33 ± 0.24		2.33 ± 0.24		2.42 ± 0.23		2.36 ± 0.23	

FEV/FVC(%) -Butchers	77.33 ± 10.24		76.66 ± 10.50		74.12 ± 10.54		75.07 ± 10.06	
FEV/FVC(%) -AS	88.25 ± 4.16		88.25 ± 4.16		88.93 ± 2.58		88.25 ± 4.16	

*Statistically significant difference (*P* < 0.05) between the butchers and administrative staff (AS). PEV = peak expiratory volume; FEV = forced expired volume; FVC = forced vital capacity.

### Concentration of Urinary 1-hydroxypyrene (1-OHP), Phenolic Compounds and Some Heavy Metals

Table 6 shows that the concentration of 1-hydroxypyrene (1-OHPyr), a PAH metabolite, in the post-shift urine samples of the butchers was significantly higher (*p* < 0.05), with mean concentration ± SD value of 0.52 ± 0.13 μg/molCret in the butchers relative to the administrative staff (0.20 ± 0.07 μg/molCret). Urinary phenol concentrations in the post-shift urine of butchers and administrative staff show that the phenol concentration of 14.26 ± 1.19 mg/l in the butchers was significantly higher (*p* < 0.05) than the 4.44 ± 1.12 mg/l found in the administrative staff. Similarly, the concentrations of zinc (test versus control: 0.91 ± 0.19 and 0.31 ± 0.28 mg/l) and nickel (test versus control: 0.11 ± 0.06 and 0.06 ± 0.02 mg/l) were significantly higher in the butchers compared to the administrative staff. The other heavy metals studied, arsenic and cadmium, were present in the urine samples in concentrations that were statistically similar (*p* > 0.05) between the two groups.

**Table 6:** Concentrations of urinary 1-hydroxypyrene, phenol and some heavy metals in butchers and administrative staff at Dei-Dei Abattoir, Abuja.

Factor	Butchers (n = 156)	Admin. Staff (n = 32)	*P*

1-OHP (μg/molCret)	0.52 ± 0.13	0.20 ± 0.07	<0.001
Phenol (mg/L)	14.26 ± 1.19	4.44 ± 1.12	<0.001
Zinc (ppm)	0.91 ± 0.19	0.31 ± 0.28	<0.001
Arsenic (ppm)	0.002 ± 0.001	0.001 ± 0.001	0.356
Cadmium (ppm)	0.26 ± 0.32	0.09 ± 0.12	0.112
Nickel (ppm)	0.11 ± 0.06	0.06 ± 0.02	0.011

## Discussion

End-of-life tyres (ELTs) find many uses, including in the defurring of animal skins and whole animals meant for human consumption. In order to cut costs, save time, and maximize profits, butchers in Nigerian abattoirs engage in this practice, despite the fact that using tyre fires in animal processing is illegal and frowned upon by regulatory agencies overseeing these abattoirs.

This study investigated the implications of this practice on the health of workers in the abattoir following chronic exposure to emissions from burning tyres utilized by butchers as described earlier. Fumes from tyres are complex mixtures and are usually difficult to quantify, especially in human exposures in occupational settings [21]. According to studies by Stellmann [22] and Mannahan [23], these complex mixtures of toxicants found in fumes from burning tyres can act to inhibit physiological pathways, elicit pronounced multiplicative effects or cause abnormalities due to the sum of individual effects. Each chemical in toxic mixtures is thought to act on multiple targets, and some of them are known to interact with other chemicals in the body (resulting in accentuation or attenuation of their toxic effects) [21]. In human exposure studies and usually in occupational settings, a more holistic approach involving the indirect evaluation of environmental data and direct measurement of human biomarkers of exposure and effect [13] is usually advised.

The results of the questionnaire administered to both groups of subjects (Table 1) to determine their sociodemographic characteristics and their degree of awareness of the health implications of using ELTs in meat processing showed that most respondents, especially the butchers, acknowledged that the use of tyres was not the healthiest option. Most of the butchers cited their inability to afford healthier alternatives as the major reason they still engage in this practice. In summary, the butchers were significantly younger, taller and had lower BMI compared to the administrative staff. During the two weeks that preceded the administration of the questionnaire, most of the butchers complained of frequent chest pain, cough, constant feeling of malaise and breathlessness following strenuous work (Table 1). Some of the administrative staff (7%) complained of breathlessness usually only after strenuous exercise. Apart from the toxic fumes to which they are marginally exposed, such symptoms may be attributable to their being more obese and older compared to the butchers.

In this study, the administrative staff served as the control group in the classical epidemiological practice of comparing the exposed and the unexposed in a study. This method is in agreement with several documented studies, especially in the area of environmental and occupational toxicology. Buchet and colleagues [24] studied coke and graphite manufacturing personnel, and as the control group, they had administrative staff from different sections of the same plant not actually involved in the coking process/activities. Many other studies comparing the effects of toxicants, especially those emanating from the burning of biomass, employed similar methods. For example, Wu and colleagues [25] studied coking workers and administrative staff from the same plant; Hu and colleagues [26] studied incinerator operators and management staff; Wang and colleagues [13] studied steel workers and office and hospital staff from the same complex.

The results of the particulate matter measurement showed a trend that was both point and time dependent. It was observed that in all the days and times sampled, point A had the highest concentration of particulate matter with a record point concentration of 5.73 μg/cm^3^ to 6.29 μg/cm^3^ recorded between the hours of 8:00 a.m. and 8.30 a.m. It could be argued that such levels of emission is to be expected given that it was the source for all the fume emissions in the abattoir. To the best of the authors’ knowledge, the health impact of exposure to tyre fire fumes utilized in the process of defurring edible animal parts has never been studied in Nigeria, thus making comparisons with local data impossible.

Emissions at points B and C were significantly (*p* < 0.05) lower than what was measured at point A (Table 3). Health effects of PM are more pronounced with exposure to PM of diameters of 2.5 μm and lower because of their deposition characteristics [7]. The smaller the aerodynamic diameter of the particle, the deeper it is deposited in the lungs, where the health impact is most felt. Studies have implicated the rise in chronic obstructive pulmonary diseases (COPD) and other pulmonary obstructive ailments to exposure to fumes from incendiary sources and usually in occupational settings or from occupational sources affecting the surrounding environment [27]. Other health problems associated with PM exposure reported by the respondents included breathlessness, constant feeling of malaise, et cetera, which are all classical symptoms of COPD. Apart from COPD and other forms of pulmonary impairments, PM is known to have PAHs, VOCs, heavy metals, organic and biogenic substances adsorbed onto it [7].

Working under these conditions, the butchers are exposed to these fumes not only via inhalation, but also through the skin. None of the butchers was adequately geared, because they could not afford protective clothing (Figure 1). Studies have shown that subjects are usually more exposed through absorption via the skin than through other routes [28]. Butchers in Dei-Dei Abattoir were most likely exposed to these chemicals mostly through dermal and inhalation routes and slightly through the oral route as some of them manage to eat as they do their work.

Not only was the point of sampling of major importance in estimating the level of exposure and, indirectly, the enormity of the deleterious health effects as a result of exposure to tyre fumes, but also time was of paramount importance [29]. From the study, it was observed that the highest concentrations of particulate matter were measured between the hours of 8:00 a.m. and 8:30 a.m. across the points (Table 3). This actually coincided with the time when most of the animals had been slaughtered and the parts sent out for defurring. The major work in the abattoir, especially in the defurring section, is at its highest point within the given time range.

Naphthalene, anthracene, fluoranthane, pyrene and benzo-a-pyrene had significantly higher concentrations at point A compared to points B and C. PAHs usually retain the chemical characteristics of the source biomass; hence, PAH mixtures are inherently different depending on whether the source of production is petrogenic or pyrogenic [30]. In this study, the PAHs detected were consistent with some of the data reported by Downard and colleagues [31]. Most of the PAHs detected in the abattoir were in the 4–6 carbon range. The translocation of the resultant plume emanating from point A depended largely on the physicochemical properties of the PAHs (molecular weight, density, photosensitivity, etc.) and the prevailing atmospheric conditions (wind, rain, etc.) to determine the concentration of PAHs at 10 meters, 100 meters, 1 kilometer from the point source [1].

Apart from other urinary metabolites indicative of PAH exposure, 1-hydroxypyrene remains the most assayed. This is because PAH exposures occur as a mixture in nature [32], and pyrene, the PAH precursor of 1-hydroxypyrene is usually found in all PAH exposure studies. Again, 1-OHP seems not to be affected by most interferences that serve as confounders in this situation [33] and might more accurately reflect the overall exposure to PAHs, taking into account all absorption routes [21,33]. 1-OHP is also a good indicator for mutagenic activities as reported in a study by Jongeneelen *et al.* [34] The 1-OHP concentration in butchers in this study was 0.52 ± 0.13 μg/molCret as against 0.20 ± 0.07 μg/molCret in the administrative staff. It is important to note that exposure to PAH through other possible sources is likely minimal as the respondents had essentially similar sociodemographic characteristics (Table 1). It is therefore safe to assume that the 1-OHP concentration measured in post-shift urine of butchers and the administrative staff was mainly due to occupational exposure to pyrene in the emissions.

In the same vein, of the four heavy metals (zinc, arsenic, nickel and cadmium) determined in the post-shift urine of the respondents, zinc and nickel were significantly higher in the butchers when compared to the administrative staff (Table 6). Arsenic was barely detectable, with almost similar concentrations in both the butchers and the administrative staff. This is suggestive of nonoccupational sources of exposure to arsenic, possibly through the consumption of contaminated food materials. Cadmium concentrations in the post-shift urine of butchers were statistically similar to those of the administrative staff. Heavy metals are persistent in nature, and health effects once exposed to them depend largely on the age and nutritional status of the individual [35].

The four metals studied are known to be incorporated in the manufacture of tyres [6] and are known to adsorb to particulate matter, especially PM_2.5_, produced during the pyrolysis of tyres [6,7]. Because of this, heavy metals can be inhaled into the lungs along with other volatile organic compounds (VOCs). Suspended heavy metals in the fumes can also be absorbed through the skin and consumed orally via unwashed fruits, vegetables and other food sources accessible in and around the impact zone of these fumes. Heavy metal toxicity has been studied in various fields and situations have been implicated (alongside PAHs) in the increase in the incidence of DNA strand breaks, which increase susceptibility to cancers [36]. Though the mechanism for this is still poorly understood, Mukherjee *et al.* [37] proposed that particle bound metal-PAH complex elicits the formation of reactive oxygen species (ROS), which through Fenton reaction can lead to the oxidation of the major classes of macromolecules, especially the DNA.

High urinary phenol concentration could be a latent pointer to the chronic buildup and/or onset of health issues of endocrinological origin. Butchers in this study had urinary phenol concentrations significantly elevated relative to the concentration in the administrative staff. The measurement of urinary phenol concentration was aimed at correlating the impact of exposure to PAHs, VOCs, heavy metals and particulate matter on the health of butchers and administrative staff. Results confirm that higher exposure to the above-named toxicants in butchers induced higher excretion of urinary phenol and consequently points to an imbalance in the endocrine health of the butchers. High values of urinary phenolics are usually indicative in some cases of hyperthyroidism, diabetes mellitus, nephrosis, obesity, hypertension or catecholamine-producing tumours, especially pheochromacytoma and neuroblastomas [21], and in this study it points to exposure-related events.

Chronic exposure to fumes from burning tyres is seen to have affected the lung function of the butchers, demonstrated by reported breathlessness and cough and other more serious indications. Spirometric measurement of the PEV showed that the butchers were able to expire significantly lower volumes of air compared to the administrative staff. FEV and FVC were significantly higher in the butchers than in the administrative staff. According to the Global Initiative for Chronic Obstructive Disease (GOLD) [38], an FEV/FVC ratio less than 80% is indicative of primary lung impairment; whereas, a ratio of less than 70% is indicative of obstructive pulmonary impairment. The FEV/FVC ratio in this study was >80% for the administrative staff but <80% in the butchers. This implies that the lung function of the administrative staff was not as impaired as that of the butchers. The butchers may be experiencing a gradual hardening of the alveoli and other lung tissues [39], making it increasingly more difficult for their lungs to remain pliable enough to perform its basic function. Chronic exposure to tyre fire fumes consisting of PM, PAHs, heavy metals and VOCs could impair alveolar–arterial oxygen balance, cause ventilation/perfusion mismatch, diffusion limitation, hypoventilation and, ultimately in chronic exposures, lead to asthmas and chronic obstructive pulmonary diseases (COPD) [40]. Peluso *et al.* [27] reported a positive correlation between increase in COPD and exposure to fumes from burning biomass.

## Conclusion

In conclusion, the results of this study show the practice of using burning tyres to defur edible animal parts is associated with many deleterious health effects for butchers who are directly exposed as compared to the administrative staff. Deleterious health effects range from pulmonary complications (impairment of lung function) to skin problems, malaise and possibly genotoxic events. A simple and cheap alternative for defurring animals should be provided by officials, and the use of personal protective equipment should be encouraged.
